# Public views on the Covid‐19 immunity certificate: A scoping review

**DOI:** 10.1111/hex.13589

**Published:** 2022-09-28

**Authors:** Serena Barello, Marta Acampora, Michele Paleologo, Lavinia Schiavone, Gloria Anderson, Guendalina Graffigna

**Affiliations:** ^1^ EngageMinds HUB—Consumer, Food and Health Engagement Research Center Università Cattolica del Sacro Cuore Milan Italy; ^2^ Faculty of Psychology Università Cattolica del Sacro Cuore Milan Italy; ^3^ Department of Psychology Università Cattolica del Sacro Cuore Milan Italy; ^4^ Department of Biomedicine and Prevention University of Rome Tor Vergata Rome Italy; ^5^ Faculty of Agriculture, Food and Environmental Sciences Università Cattolica del Sacro Cuore Cremona Italy

**Keywords:** Covid‐19, Covid‐19 immunity certificate, health certificate, immunity passports, public opinion

## Abstract

**Introduction:**

Already in its first implementation, the introduction of the Covid‐19 immunity certificate has generated some debate among the public. This debate might be a hindrance to the effective realization of this policy. This study aimed to systematically review published research evaluating public feeling of the Covid‐19 immunity certificate policy measure and to find which factors might influence its acceptance.

**Methods:**

We followed the scoping review methods manual by the Joanna Briggs Institute. We included studies with no time limits that presented novel data, and no exclusions have been made based on study design. We excluded articles that presented just expert opinions.

**Results:**

We found and reviewed 17 articles. The included studies were conducted in two main countries (the United Kingdom and Switzerland), with the rest from Israel, Italy, Spain, Germany, Australia, Taiwan and China. Both qualitative and quantitative studies were included, and nonrepresentative samples were mostly used to explore the public feeling about the Covid‐19 immunity certification. The included studies showed that public views on immunity certification are quite contradictory and influenced by age, gender, ethnicity, political orientation and attitudes towards Covid‐19 vaccination. The topic more often addressed by the included studies was the public's views on the positive and negative implications of the Covid‐19 immunity certificate in terms of ethical, legal and behavioural consequences of this measure.

**Conclusion:**

The varying acceptance rates are notable and may partly be linked to differences in demographics, Covid‐19 concerns and ideological beliefs, as seen in other health‐related tracking policies. Moreover, dominant factors behind the (un)success of this policy are complex and entangled with the cultural and political dimensions rather than being just technical. For this reason, it is important to expand psychosocial research to better understand the concerns behind health certifications and allow planning of culturally based and ethically sound suitable strategies. This would be very relevant to increasing public approval and compliance with this public health measure.

**Patient or Public Contribution:**

This does not apply to our work as it was a review paper.

## 1 INTRODUCTION

Since the start of the Covid‐19 vaccination campaigns, vaccine hesitancy has been recognized as one of the foremost barriers to their effectiveness.^1^, ^2^, ^3^ As the Covid‐19 pandemic continues and global vaccination campaign does not seem to be enough to effectively control the virus, many countries proceed to (re)instituting lockdowns and other restrictive public health measures to manage the spread of the disease.^2^ Governments are simultaneously struggling with ways to save people from some of these restrictions. One nonpharmaceutical initiative that is being carried out is immunity certifications (also called the ‘Covid‐19 immunity certificate’) to exempt vaccinated people and/or those with evidence of immunity (depending on the country in which this measure has been applied) from some limitations.^3^

The Covid‐19 immunity certificate is a measure that aims to relax restrictions for individuals who are proved to be immune and, at the same time, to contain the contagion to recover social, cultural and working activities.^4^

When compared with other incentives to tackle vaccine hesitancy, immunity certification looks like a very promising concept, as it gives incentives to people to get vaccinated without imposing a mandatory behaviour (i.e., mandatory vaccination).^5^ However, even during its first adoption, this measure raised concerns^3^, ^6^, ^7^ and generated debate among the public since it may be used as a tool for possible discrimination based on someone's health status.^8^, ^9^, ^10^ For these reasons, opposition movements started to grow in many countries.^11^, ^12^, ^13^

Although attitudes towards immunization certificates have been investigated by some studies, to our knowledge, this evidence has not been systematically reviewed. Therefore, this study aimed to systematically review research evaluating public attitudes towards the Covid‐19 immunity certificate and to identify what factors might influence its acceptance.

## 2 METHODS

We followed the Preferred Reporting Items for Systematic reviews and Meta‐Analyses extension for Scoping Reviews guidelines and the scoping review manual developed by the Joanna Briggs Institute.^14^ According to this manual guideline, the present review was performed through the following steps.

### 2.1 Stage 1: Formulating the research question

Our scoping review aimed to answer the following question: ‘what evidence exists to describe how the public perceives the Covid‐19 immunity certificate and what are the influencing factors of such attitudes in ways that could inform public messaging that introduces citizens to this measure’.

### 2.2 Stage 2: Identifying relevant studies

We searched a broad range of databases: PubMed, Scopus, PsycInfo, Web of Science and Google Scholar. Moreover, four preprint databases, SocArXiv, MedRXiv, PsyRXiv and SSRN, were included in the search. The selection of search terms was performed by a researcher experienced in systematic reviews (G. A.). The following search string was adopted to collect relevant records: ((Persons OR Citizen OR Adults OR ‘Young adults’ OR Elderly OR ‘older people’ OR ‘Minority groups’ OR ‘Healthcare workers’) AND (‘Green Pass’ OR ‘Vaccine passport’ OR ‘Vaccine passports’ OR ‘Vaccination certificate’ OR ‘Immunity certificate’ OR ‘Immunity passport’ OR ‘Health passport’ OR ‘Health certificate’ OR ‘Health license’ OR ‘Health code’ OR ‘Health pass’ OR ‘Immunity‐based license’ OR ‘Mandatory vaccination’ OR ‘Mandatory immunization’ OR ‘Mandatory immunisation’ OR ‘Compulsory vaccination’)) AND (Attitude OR Attitudes OR ‘Attitude to health’ OR ‘Health attitudes’ OR Belief OR Beliefs OR Acceptance OR Behavior OR Behaviour OR Behaviors Or Behaviours OR Opinion OR Opinions OR Sentiment OR Sentiments OR Willingness OR motivation OR disposition OR inclination). We extracted and uploaded all results, except those from Google Scholar, into Zotero, a software that facilitates systematic or scoping reviews.

### 2.3 Stage 3: Study selection

The following inclusion criteria were used:

1. Participants: Studies were included if they investigated either public attitudes towards the Covid‐19 immunity certificate or the factors influencing public attitudes towards this measure.
2. Concept under investigation: The action or process of providing an official document that grants access to activities based on (a) negative test results for Covid‐19, or (b) vaccination against Covid‐19, or (c) proof of infection then recovery, or (d) antibody testing.
3. Outcomes: Public attitudes towards the Covid‐19 immunity certificate; factors affecting the acceptance of the Covid‐19 immunity certificate.
4. Study design: Studies were included if presenting novel data and no exclusions were made based on study design.
5. Characteristics: Articles published with no time limits.
6. Language: Only English‐ and Italian‐language studies were included in the search.

Moreover, we expanded search of the references of the selected articles and grey literature for comparable search terms, which allowed us to add a small number of studies judged to be relevant that were not found in the search. Three authors (M. A., M. P., L. S.) independently reviewed the titles and abstracts of all retrieved articles and made decisions on inclusion by consensus according to the predefined inclusion and exclusion criteria. One author (S. B.) reviewed all articles selected for full‐text review, and two authors (M. A., M. P.) each reviewed half of the total study group selected for full‐text review.

### 2.4 Stage 4: Charting the data

A standardized data extraction sheet was developed and completed by the authors. Three investigators (M. A., M. P., L. S.) populated the data extraction sheet.

### 2.5 Stage 5: Collating and summarizing the results

We conducted a narrative/thematic analysis of the research. The main characteristics of the studies included are reported in Table 1. Findings about the factors that influenced Covid‐19 immunity certificate public views were analysed and are presented in Table 2.

**Table 1:** Main characteristics of the included studies (*n* = 17)

Authors, year of publication	Country	Aim	Population, sample (*N*)	Study design	Methods for data collection	Data collection period
Aranzales et al. (2021)^15^	Australia	To probe the scientists' opinions on immunity certificates as a potential instrument to lessen the impact of the Covid‐19 crisis.	International scientist, (*N* = 12,738)	Quantitative	Survey	4 May to 3 June 2020
Arias‐Oliva et al. (2021)^16^	Spain	To study the impact of ethical judgements on user attitudes towards using vaccine passports based on a Multidimensional Ethics Scale (moral equity, relativism, egoism, utilitarianism and contractualism).	General Spanish population, (*N* = 400)	Quantitative	Survey	16 April 2021 to 29 April 2021
De Figueiredo et al. (2021)^17^	UK	To understand if the vaccine passports are likely to increase inclination to accept a Covid‐19 vaccine.	General UK population, (*N* = 17,611)	Quantitative	Survey	9–27 April 2021
Fargnoli et al. (2021)^18^	Switzerland	To identify arguments in favour of and against the possible implementation of immunity certificates related to Covid‐19 in Switzerland.	General Swiss population living in Geneva, (*N* = 68)	Qualitative	Focus group and semi‐structured interviews	July–November 2020
Gallè et al. (2021)^19^	Italy	To assess the acceptance of Covid‐19 vaccination in a sample of older adults and to assess the attitude towards the adoption of the Covid‐19 immunity certificate.	Older adults (≥65) from southern Italy (Apulia), (*N* = 1041)	Quantitative	Survey	June–August 2021
Garrett et al. (Preprint)^20^	Australia, Japan, Taiwan, Germany, Spain and the United Kingdom	To assess attitudes towards the introduction of immunity passports.	General international population, (N = 12,944)	Quantitative	Survey	April–May 2020
Green et al. (2021)^21^	Not specified	To examine how perceived recovery from Covid‐19 and the concept of immunity passports influence people's intentions to engage in behaviours aimed at reducing the spread of Covid‐19.	General international population, (*N* = 394)	Quantitative	Survey	2020
Hall et al. (2021)^22^	USA	To figure out public views regarding government and private conferral of immunity privileges.	General US population, (*N* = 1315)	Quantitative	Survey	June 2020
Hu et al. (2021)^23^	China	To examine the Chinese public's attitudes to the so‐called Covid‐19 vaccination passport and factors contributing to their viewpoints.	General Chinese population, (*N* = 2038)	Quantitative	Survey	April 2021
Khan et al. (2022)^24^	Not specified	This study is aimed at understanding the positive and negative discourse surrounding the Covid‐19 passport system.	Tweets originated from personal accounts, followed by media organizations, media‐related personalities, politicians and the travel industry (*N* = 512 tweets)	Mixed methods (qualitative and quantitative) on web contents	Twitter	24 May 2021 to 17 June 2021
		Based on sound theoretical foundations of the Health Belief Model and the Theory of Planned Behaviour, this study offers an exploratory analysis of Twitter data about the Covid‐19 passport on Twitter.				
Lewandowsky et al. (2021)^8^	The United Kingdom	To probe people's attitudes towards tracking technologies and immunity passports to understand which aspects are considered acceptable and which might be opposed because of their privacy implications.	General UK population, (*N* = 1446)	Quantitative	Survey	16 April 2021
Mayssam et al. (2020)^25^	Switzerland	To assess the social and individual views of immunity and vaccination certificates	Swiss general population living in Geneva, (*N* = 1425)	Quantitative	Survey	May–June 2020
Nehme et al. (2021)^26^	Switzerland	The study aimed at assessing the public view of Covid‐19 vaccination certificates as well as potential differences between individuals.	General Swiss population living in Geneva, (*N* = 4067)	Quantitative	Survey	17 March to 1 April 2021
Porat et al. (2021)^27^	The United Kingdom and Israel	To investigate whether people's willingness and motivation to get vaccinated relate to their psychological needs and how vaccine passports might affect these needs.	General UK and Israel population, (*N* = 1358; 681 from the United Kingdom; 677 from Israel)	Quantitative	Survey	10 May to 14 May 2021
Shmueli (2022)^28^	Israel	To assess the Israeli public's intention to get vaccinated at once after the Covid‐19 vaccine became available and to determine the role of incentives beyond sociodemographic, health‐related and behavioural factors in predicting this intention.	General Israel population, (*N* = 461)	Quantitative	Survey	22 December 2020 to 10 January 2021
Spitale et al. (Preprint)^12^	Italy	To understand and describe the concerns of the anti‐ Covid‐19 immunity certificate by individuals in Italy, the main arguments of discussion and their characterization.	Telegram chat of the general Italian population against the Covid‐19 immunity certificate (two groups of chats: no‐immunity certificate group of Italian university students and generic no‐immunity certificate group)	Mixed‐methods approach (qualitative and quantitative) on web contents	Telegram	9 September 2021
Wang et al. (2021)^29^	China	To examine the effects of a ‘Health code’‐based vaccine mandate on willingness to be vaccinated for Covid‐19	General Chinese population vaccine‐hesitant, (*N* = 873)	Quantitative	Experimental design	August 2020

**Table 2:** Narrative synthesis of the main topics associated with the public attitudes towards the introduction of the Covid‐19 immunity certificate reported by the included studies (*N* = 17)

Author (year of publication)	Country	Public attitudes towards the Covid‐19 immunity certificate	Factors shaping public attitudes towards the Covid‐19 immunity certificate
Aranzales et al. (2021)^15^	Australia	Scientists perceive immunity certificates as favourable for public health (50.2%) and the state of the economy (54.4%), while one‐fifth (19.1%) and one‐sixth (15.4%) disagree.	Ethnicity Gender Political orientation Scientific fields
		Scientists stipulate some concerns about fairness (36.5%) and inequality (22.4%) arising from the implementation of immunity certification.	
Arias‐Oliva et al. (2021)^16^	Spain	Immunity passport acceptance increase when people express a positive evaluation of moral equity, egoism and utilitarianism about this measure.	Moral attitude
de Figueiredo et al. (2021)^17^	The United Kingdom	A large minority of respondents reported that vaccination passports for domestic use (public houses, restaurants, nightclubs, sporting events) or international travel would make them no more or less inclined to accept the Covid‐19 vaccine, and a sizeable minority of respondents also state that they would accept the Covid‐19 vaccine and that vaccine passports would make them more inclined to get vaccinated.	Age Ethnicity
		A majority of the UK public believe that immunity passports are a good idea (59.8%). More respondents believe that passports do not infringe on personal liberties (41.1%).	
Fargnoli et al. (2021)^18^	Switzerland	Few participants considered immunity certificates based on serological testing as an acceptable public health measure.	N/A
		On the one hand, participants reported some benefits related to the immunity certificate, such as increasing intentions to get vaccinated, gain medical knowledge and protection in a certain context involving leisure‐ or work‐related activities.	
		On the contrary, some harms were also reported: discrimination, counterfeiting, incitement to self‐infection, invasion of the private sphere, violation of personal integrity and violation of medical secrecy were perceived as the major risks.	
Gallè et al. (2021)^19^	Italy	33.3% of participants were favourable to the adoption of the Covid‐19 immunity certificate.	Vaccination status
		Regarding the comparison between the period before and after the mandatory adoption of the Covid‐19 immunity certificate, the following statistically significant result was obtained: The percentage of those who were favourable to the Covid‐19 immunity certificate changed from 44.9% to 16.2%.	
		It is possible to observe a significantly higher percentage of people nonfavourable to mandatory vaccination among vaccinated individuals who took part in the study after the adoption of the Covid‐19 immunity certificate measure.	
Garrett et al. (Preprint)^20^	Australia, Japan, Taiwan, Germany, Spain and the United Kingdom	Immunity passport support was moderate to low, ranging from 51% in the United Kingdom and Germany, down to 22% in Japan.	Gender Attitudes towards the Covid‐19 immunity certificate Covid‐19 concern Political orientation
Green et al. (2021)^21^	N/A	Immunity passports were mostly supported when participants were exposed to information presented with sensitivity towards the current scientific consensus concerning infection‐acquired COVID‐19 immunity.	Evidence‐based policy introduction
Hall et al. (2021)^22^	USA	45.2% of respondents supported immunity privileges, with slightly more favouring private certificates than government passports. Support was greater for using passports or certificates to enable return to high‐risk jobs or attendance at large recreational events than for returning to work generally.	Ethnicity Gender Attitudes towards the Covid‐19 immunity certificate
Hu et al. 2021^23^	China	The Chinese people had favourable opinions on the passport.	Income Political orientation Attitudes towards Covid‐19 vaccination Attitudes towards the Covid‐19 immunity certificate
		An average of 29.91% agreed or agreed with the statements related to vaccine passports, while only 8.28% disagreed or disagreed with them.	
Khan et al. (2022)^24^	N/A	Most Twitter users indicated favourable attitudes (61%, *n* = 283) towards the Covid‐19 passport compared to 39% of tweets (*n* = 182) stating negative sentiment (antipassport) towards the Covid‐19 passport.	Trust in government Conspiracy theory inclinations Covid‐19 immunity certificate information literacy Self‐efficacy
		Travel emerged as the dominant theme. There were 137 tweets (29.5%) that referred to travel as a benefit of having some sort of a COVID‐19 passport, followed by social (4.3%) and economic benefits (3.9%).	
		Lack of consensus on a common standard (17%; *n* = 79), digital divide (6%; *n* = 3), privacy considerations (2.6%; *n* = 12) and personal freedoms and human rights (9.9%; *n* = 46) stood as the most salient barriers.	
Lewandowsky et al. (2020)^8^	The United Kingdom	Most people did not object to the idea of passports, with the concern being low on average and more than 60% of people wanting one for themselves to varying extents. There were, however, around 20% of respondents who considered passports to be unfair and who opposed them completely.	Age Attitudes towards the Covid‐19 immunity certificate Covid‐19 concern Trust in government
Mayssam et al. (2020)^25^	Switzerland	About 80% of participants agreed that knowing one's serology status would lead to a change in one's behaviours. In the event that the presence of antibodies correlated with immunity, 60% of participants reported that certificates should be offered to the general population. The results showed variations in attitudes towards certificates depending on the context (73% agreed on certificates' utility for travel, 72% for entering a country and 32% for the right to work). Provided an effective vaccine was available, 55% of participants agreed that vaccination should be mandatory and 49% agreed that a vaccination certificate should be mandatory.	Age Educational level Gender
		About 68% reported a potential risk of discrimination and 28% reported a risk of deliberate infection.	
Nehme et al. (2021)^26^	Switzerland	About 61.0% of participants agreed or strongly agreed that a vaccination certificate was necessary for certain contexts and 21.6% believed that there was no context where vaccination certificates should be presented. Contexts where most participants perceived a vaccination certificate should be presented included jobs where others would be at risk of COVID‐related complications (60.7%), jobs where employees would be at risk of getting infected (58.7%) or to be exempt from quarantine when travelling abroad (56.0%).	Age Educational level Gender Income Professional status Vaccination status
Porat et al. (2021)^27^	The United Kingdom and Israel	Vaccine passports received considerable backlash and criticism, with citizens and healthcare experts seeing them as coercion and against individual autonomy and freedom of choice. Vaccine passports are perceived as frustrating psychological needs, particularly people's sense of autonomy.	Autonomy frustration Need frustration
Shmueli (2022)^28^	Israel	Higher support towards immunity certificates has been demonstrated to be predictive of people's intention to get vaccinated against Covid‐19.	Vaccination status
Spitale et al. (Preprint)^12^	Italy	Messages related to the rule vaccine had a 96.26% probability to depict negative sentiment, a particularly high probability also when compared with negativity for Covid‐19, Freedom and Covid‐19 immunity certificate opposition chats (90%, 88% and 85%, respectively), thus providing strength to the hypothesis that vaccine scepticism is the primary reason to oppose the Covid‐19 immunity certificate.	Attitudes towards Covid‐19 vaccination Conspiracy theory inclination
		Harms related to immunity certificates revolve around legal aspects and the limitation of personal freedom.	
Wang et al. (2021)^29^	China	For individuals with higher educational attainment, willingness to vaccinate for Covid‐19 in China will increase if the immunity certificate is used to restrict unvaccinated individuals from public spaces.	Vaccination status

## 3 RESULTS

### 3.1 Search results

**Figure 1:**
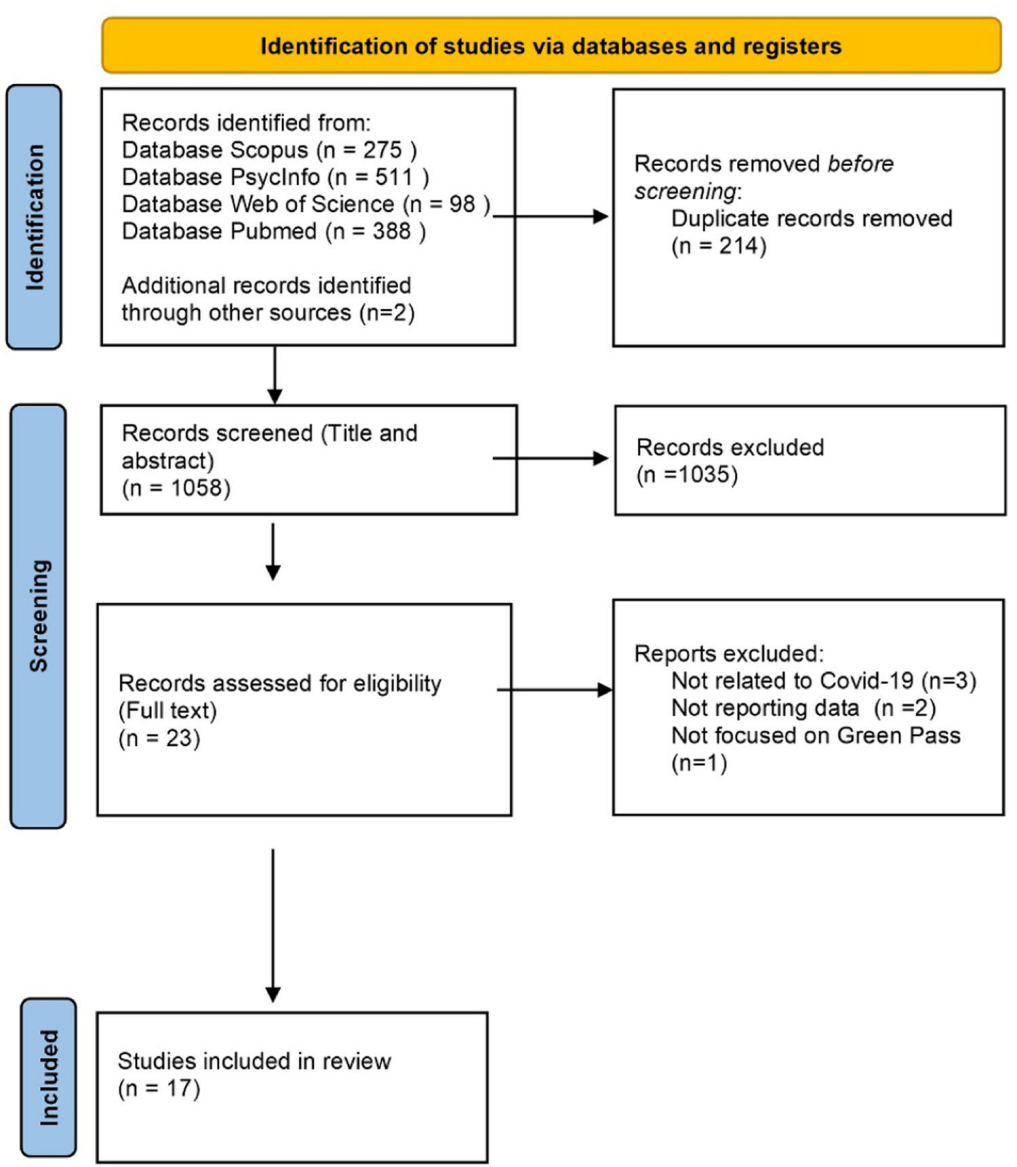
PRISMA flow diagram. PRISMA, Preferred Reporting Items for Systematic reviews and Meta‐Analyses

Titles and abstracts of 1272 records were retrieved. After the removal of duplicates, 1058 records were examined. The screening process is summarized in Figure 1. Based on the assessments of titles and abstracts, 1035 records were excluded because they did not explore qualitative or quantitative data about public attitudes towards the Covid‐19 immunity certificate or variables influencing the public views on this public health measure. Of the 23 fully read papers, 15 fulfilled the inclusion criteria. After the backward reference tracking, grey literature search and preprint database search, 2 further preprint studies were included,^9^, ^23^ and the final review included 17 papers.

### 3.2 Study characteristics

#### 3.2.1 Country of study and year of publication

Most studies were conducted in the United Kingdom and Switzerland—respectively, four^8^, ^17^, ^20^, ^27^ and three^18^, ^25^, ^26^—and the remaining were from Israel,^27^, ^28^ Italy,^12^, ^19^ Spain,^16^, ^20^ Australia,^15^, ^20^ Japan,^20^ Taiwan,^20^ Germany,^20^ United States of America and^22^ China.^23^, ^29^ Only one study did not report the country of data collection because it was conducted on Twitter.^24^ The studies were published between 2020^25^ and 2022.^24^, ^28^

#### 3.2.2 Study populations and samples

All included studies involved a population of adults outside of healthcare settings. The majority of studies involved the general population,^8^, ^16^, ^17^, ^18^, ^20^, ^21^, ^22^, ^23^, ^25^, ^26^, ^27^, ^28^ one study involved a sample of international scholars,^15^ one study involved older adults,^19^ one study involved university students attending no Covid‐19 immunity certificate Telegram chat^12^ and one study involved vaccine‐hesitant people.^29^ Just one study sampled 512 tweets that originated from personal accounts, followed by media organizations, media‐related personalities, politicians and the travel industry.^24^ Almost all studies used nonprobability sampling techniques, in particular, convenience samples. Just six studies^8^, ^16^, ^20^, ^22^, ^25^, ^27^ used random representative samples. For quantitative studies, samples varied from 394^21^ to 17,611 participants.^17^ For the only one retrieved qualitative study, the sample included 68 participants.^18^

#### 3.2.3 Study methods

Fourteen studies used quantitative methods, one study used qualitative methods and two studies adopted a mixed‐methods design applied to the analysis of web conversation. The majority are quantitative cross‐sectional surveys (*n* = 14). For qualitative research, the included study adopted focus groups and interviews. Studies on web conversation analysis adopted a mixed‐methods approach.

#### 3.2.4 Study timing

Most studies (*N* = 10) were conducted in 2021. Data collection was mainly concentrated in April 2021.^8^, ^16^, ^17^, ^23^, ^26^ Just seven studies collected data in 2020.^15^, ^18^, ^20^, ^21^, ^22^, ^25^, ^29^

### 3.3 Main findings

In the following paragraphs, we present a narrative synthesis of the main topics associated with the public attitudes towards the introduction of the Covid‐19 immunity certificate. The topic more often addressed was the public views on its positive and negative implications in terms of its ethical, legal and behavioural consequences. Furthermore, we identified the main factors influencing public attitudes towards the Covid‐19 immunity certificate. All the results are summarized in Table 2.

Most studies reported that the majority of respondents are favourable to the adoption of immunity certificates.^8^, ^15^, ^17^, ^23^, ^24^, ^25^, ^26^ One study reported that 49% of the population think that immunity certification should be mandatory.^25^ However, limited research found the opposite result. For instance, Gallè et al.^22^ found that only 33% of respondents were favourable of this measure. Even Garret et al.^20^ found that immunity certificate support was moderate to low, ranging from 51% in the United Kingdom and Germany and down to 22% in Japan. Other studies reported opinions that support immunity certificates only for specific work‐related contexts^18^ and not for attending large gatherings or social venues.^26^ Other research reported a favourable public opinion towards the adoption of an immunity certificate for crossing international borders, taking a plane or avoiding quarantine related to travel.^25^

### 3.4 Perceived benefit

Arguments in favour of the Covid‐19 immunity certificate were related to some individual and collective benefits they could provide.

Regarding the individual benefits, the included studies highlighted that there are some main reasons behind the public positive attitudes towards the certificate. First, the introduction of an immunity certificate makes people feel less vulnerable to taking Covid‐19 because it may guarantee to attend safer places^8^, ^18^; then, self‐protection from Covid‐19 infection through the use of immunity certification was another mentioned motivator for this measure acceptance.^8^, ^20^ Moreover, this public health measure allows people to return to normal, prepandemic activities (travelling, leisure, work‐life, public events….) and to come back also to high‐risk jobs with less risk of contagion.^22^ Regarding the Covid‐19 vaccination issue, an immunity certificate is perceived as a valuable incentive to enhance citizens' motivation or willingness to get vaccinated.^12^, ^23^

Regarding the collective benefits, the included studies highlight the following. First, some studies underlined the role of immunity certifications in protecting public health and the most vulnerable categories of citizens (i.e., healthcare workers, older people, individuals with fragile health conditions, children…).^15^, ^18^, ^26^ Other studies found that citizens who agreed with the immunity certification measure saw it as a policy tool to reduce the economic and societal burden of the pandemic because it could help the economy recover and ensure the resumption of work activities at full capacity.^15^, ^18^, ^22^, ^24^, ^26^

### 3.5 Perceived harms

From the individual perspective, not everyone supports the use of the Covid‐19 immunity certificate. Arguments against the implementation of immunity certificates were discussed around six main potential negative implications mostly related to the potential risk of increasing inequalities about the costs and access to tests and certificates. Immunity certification seems to raise some ethical aimed societal concerns. Evidence from different studies showed how some individuals are opposing this proposal from an ethical perspective, considering potential problems, such as the risk of discrimination,^15^, ^18^ violation of liberties,^12^, ^18^ falsification^18^ and infringed data privacy,^18^, ^24^, ^25^ limited freedom of movement^17^ and negative impact on behaviours.^25^

Classifying people based on their COVID‐19 situation could lead to discrimination between the vaccinated and the unvaccinated. Moreover, some authors suggested the risk of work‐related discrimination in terms of reduced employment opportunities for those who do not have immunity certificates.^18^, ^25^ Freedom of movement is another concern that could become a problematic issue, especially for people who would not be able to take the vaccine because of health constraints.^16^ This would imply that individuals not yet vaccinated will not be able to go on international travel or may suffer domestic and local mobility restrictions.^16^ Immunity certificates were also perceived as an invasion of the private sphere and a violation of personal integrity. The public reported worries about the privacy of their health data. The risk of the violation of medical secrecy was also often highlighted.^18^

The economic value of the immunity certificate was also reported as an incentive to expose oneself to catch the disease, often expressed as an encouragement to take part in ‘corona parties’. From a societal and ethical perspective, this collateral harm was deemed intolerable.^18^

### 3.6 Factors influencing public attitudes

#### 3.6.1 Sociodemographic characteristics shaping public attitudes towards the Covid‐19 immunity certificate

Nine studies reported the sociodemographic factors associated with public attitudes towards the immunity certificate. Age,^8^, ^17^, ^25^, ^26^ education level,^18^, ^25^, ^26^ gender,^15^, ^20^, ^22^, ^25^, ^26^ ethnicity,^15^, ^17^, ^22^ income status^23^, ^26^ and professional status^26^ were the most common significant factors reported. White individuals and older people who have high‐income status were generally more likely to report positive attitudes towards the vaccines. The variable of level of education seems to be more controversial in its impact on attitudes. More educated people have been found to be more sceptical of and worried about the Covid‐19 immunity certificate due to possible risks of discrimination in some studies.^18^, ^25^, ^29^ In other studies, more educated individuals have been found to be more inclined to this measure when it is related to attending work settings where there is a high risk of infecting vulnerable people.^26^ Most of the studies reported that men were more willing to accept the Covid‐19 immunity certificate than women.^15^, ^22^, ^25^, ^26^ Regarding ethnicity, one study found that Hispanic people or individuals belonging to ethnic minorities are less prone to accept the Covid‐19 immunity certificate.^22^ Finally, some studies revealed that professional status might be an influencing factor in the public attitude towards the Covid‐19 immunity certificate.^25^, ^26^ For instance, working people seem to be less favourable than unemployed people or students.^25^ reported that managers are more inclined to accept Covid‐19 immunity certificates when connected to travel or to be exempt from quarantine when travelling abroad.

#### 3.6.2 Individual factors shaping public attitudes towards the Covid‐19 immunity certificate

Several individual factors influencing public attitudes were reported in 14 studies. Four studies reported that positive attitudes towards the Covid‐19 immunity certificate seem to be the strongest predictor of its behavioural acceptance.^20^, ^22^, ^23^ On the contrary, scepticism towards tracking measures significantly diminishes citizens' openness to the measure of the Covid‐19 immunity certificate.^8^ Moreover, how immunity passports can benefit oneself in terms of desiring a passport and being willing to infect oneself to gain an immunity certificate were all factors positively predictive of passport support.^20^ Furthermore, some studies found that personal beliefs about vaccines and vaccination status may be a major predictor of acceptance of the immunity certificate: In particular, being favourable towards getting the Covid‐19 vaccination^12^, ^23^, ^28^, ^29^ and being vaccinated^19^, ^26^, ^28^, ^29^ are predictors of the Covid‐19 immunity certificate acceptance. Moreover, few studies revealed that concerns towards Covid‐19, in terms of higher susceptibility and risk perception,^8^, ^20^ are both related to a positive judgement of the immunity certification. Additionally, people's ethical perceptions may serve as important catalysts for influencing attitudes towards immunity certificates: when citizens consider immunity certification as fair (moral equity), required by people belonging to the individual's social network—such as family, friends and colleagues—(relativism), a positive measure to attain personal objectives (egoism), also providing a social utility (utilitarianism), this becomes an acceptable mandate.^16^ Public opinion on this issue may also be influenced by political inclination and global perceptions^15^, ^20^, ^23^: according to one study, neoliberal worldviews—which consider the free market as fair and as operating best when unrestricted by government intervention—improve popular support for immunity certificates.^20^ Another study on international scientists showed that those who hold more conservative views (more right‐wing) are significantly more in favour of immunity certificates.^15^ By contrast, opposition towards immunity certificates is linked to a communitarian approach to public health, which is in line with progressive views.^15^, ^30^

A further factor that appears to influence public feelings on this issue is racial and cultural background. In one study, researchers found that while in the Western setting sentiments about the adoption of immunity certificates are more influenced by societal factors than by personal risks and advantages,^20^ in Eastern countries, the latter seems to have a more remarkable role, as a positive and robust association between subjective norms, nationalism and positive attitude to Covid‐19 immunity certificates has been found.^23^ Finally, mistrust towards government and individuals' conspiracy theory inclination are related to negative attitudes towards immunity certificates.^8^, ^24^

## 4 DISCUSSION

Over the last 2 years (2020–2022), several studies published empirical data that help to understand public's opinions regarding Covid‐19 immunity certificates among international populations, and their findings have been reviewed, analysed and summarized in this study.

The public feeling in regard to immunity certification policies has been mixed—leading to increased vaccination intention^17^ as well as protests.^13^ Even though opinions differed amongst research, the collected data indicated that public attitudes were generally supportive of the usage of immunity certificates. This discrepancy could be attributed to variations in the study population (gender, age and ethnicity). Just a few studies reported strong opposition of the public to certificates of immunity for any purpose. A significant number of the included articles reported legal and ethical concerns about immunity certifications. All these results are similar to what was discovered by other studies on public opinion towards health certification and health‐tracking technologies.^9^, ^31^, ^32^

Beyond these, the current analysis discovered other elements that further aid in comprehending public concerns. The varying acceptance rates may partly be attributed to differences in demographics, Covid‐19‐related concerns and ideological beliefs, as seen for other health‐related tracking policies.^33^ Moreover, dominant factors behind the (un)success of this policy are complex and entangled with the cultural and political dimensions rather than being just technical.^1^ Indeed, in different contexts, this certification implies different rights, restrictions and/or freedoms, based on presumed immunity‐ or infection‐based criteria. This may further explain retrieved differences in public views.^34^ For instance, our results are aligned with recent results of another study^35^ reporting that people who are more sensitive to the risk of contracting Covid‐19 also reported a greater endorsement of tracking technologies. Also, the role of political views and cultural values is coherent with other research: Scholars found that people more oriented to collectivistic values and with higher right‐wing authoritarianism were more likely to endorse tracking technologies.^35^, ^36^ Finally, we discovered that a strong predictor of immunity certification acceptance is the level of citizens' trust in government. This result is similar to what was discovered by studies investigating the determinants of vaccine acceptance.^37^, ^38^

### 4.1 Strengths and limitations of the review

To the best of the authors' knowledge, this review offers the first summary of studies in this field and offers helpful policy recommendations. There are a few restrictions. As this is a scoping review, we did not evaluate the publications' methodological quality; nonetheless, this might affect the validity of the results that were obtained. Most of the included research utilized self‐reported metrics with nonrepresentative samples. In addition, we chose to incorporate two preprint papers that were not subjected to peer review. The research only included articles written in English and Italian, which may have underrepresented public opinions in various cultural contexts.

## 5 CONCLUSION

While providing information to the public about the implications of immunity certification policies is essential, this study showed that they need to be complemented by trust‐building and culturally sound strategies to sustain their acceptance.^39^ Indeed, these policies have been currently adopted to various extents across many countries—from limiting travel to restricting admission to social venues and public events.^40^ While it is probably too early to have an overall understanding of the implications of these policies, collecting data on these experiences is helpful in not only supplying feedback to revise the implementation of such measures but also in mitigating in ‘real time’ any perceived harm. Moreover, no ‘objective’ data about the impact of immunity certification policies on vaccination rates are available in the selected studies. Further research should also evaluate this aspect to develop truly evidence‐based policies in the future as well as compare different certification policies applied to infectious diseases.

These policies also represent an interesting ‘case study’ for generating future policies in this sector and will be the testing ground for further global health strategies. The success of such a policy will be shaped by people's subjective experiences that are historically and culturally situated. As such, health certification policies should not be transferred uncritically from one culture to another. For instance, differences between low‐ and high‐income countries in the fair access to vaccination might shape individuals' feeling of immunity certification fairness. This should be carefully considered when implementing this or similar policies. To date, most of the retrieved studies focusing on public attitudes towards the Covid‐19 immunity certificate mainly focused on its ethical and legal issues. These reflections, while relevant, risk neglecting the human subjective, day‐by‐day experiences that they are rooted in. In this paper, we propose that discussions around immunity certification should include more input from the psychosocial sciences to better understand the effects of such policies on people's lives. Indeed, as suggested by other studies,^41^, ^42^, ^43^, ^44^ a number of underpinning disciplines within the social sciences, notably sociology, social psychology and anthropology, offer both theoretical insights and methodological approaches that can productively enhance the study of equity in health systems and policy planning and implementation. The knowledge of these issues and the lives with which they are associated may be further enriched by the integration of social science contributions into the development of health policy. To avoid a paternalistic approach to health planning and to promote people engagement‐sensitive initiatives,^45^ it is crucial to not reject population concerns, but rather to thoroughly analyse and investigate them when relevant. According to this viewpoint, a psychological approach to health policies might be a useful addition to the theoretical toolkit of public health ethics.

## AUTHOR CONTRIBUTIONS

This study was conceived by Serena Barello and Marta Acampora. Serena Barello, Marta Acampora, Lavinia Schiavone and Michele Paleologo collected the data. Serena Barello, Marta Acampora and Guendalina Graffigna analysed and interpreted the data and drafted the manuscript. All authors critically revised the manuscript and approved the final version.

## ETHICS STATEMENT

Ethical approval for this type of study is not required by our institute.

## Data Availability

Data are available upon request from the corresponding author.
